# Knowledge, attitude and practice regarding constipation in pregnancy among pregnant women in Shanghai: a cross-sectional study

**DOI:** 10.3389/fpubh.2024.1378301

**Published:** 2024-07-18

**Authors:** Lin Lin, Yi Yu, Weirong Gu, Rong Hu, Hao Zhu

**Affiliations:** Department of Obstetrics, Obstetrics and Gynecology Hospital of Fudan University, Shanghai, China

**Keywords:** knowledge, attitude, practice, constipation in pregnancy, pregnant women

## Abstract

**Objective:**

This study aims to investigate the Knowledge, Attitude, and Practice (KAP) pertaining to constipation during pregnancy among pregnant women in Shanghai.

**Methods:**

Demographic data and KAP scores were collected using a questionnaire. Differences across groups were analyzed using either Wilcoxon-Mann–Whitney tests or Kruskal-Wallis analysis of variance. Spearman’s correlation analysis was utilized to evaluate the relationships between KAP scores. Multivariable logistic regression analyses were conducted to identify factors that influence KAP scores.

**Results:**

Encompassing 241 individuals (46.6%) aged between 30 and 34 years, with 349 participants (67.5%) being nulliparous. The median scores for knowledge (possible range: 0–26), attitude (possible range: 7–35), and practice (possible range: 14–70) were 22 (18, 24), 26 (23, 29), and 51 (46, 56), respectively. Multivariate analysis indicated that being a medical professional (OR = 2.222, *p* = 0.043) and receiving education on constipation during pregnancy (OR = 0.432, *p* < 0.001) were significantly associated with higher knowledge scores. Factors significantly associated with practice included being aged 30–34 years (OR = 2.745, *p* < 0.001), aged 35 years and above (OR = 2.514, *p* < 0.001), working in education (OR = 2.310, *p* = 0.012), and not experiencing constipation before pregnancy (OR = 1.894, *p* = 0.001).

**Conclusion:**

Pregnant women demonstrated satisfactory knowledge, positive attitudes, and proactive practices concerning constipation during pregnancy. To further augment clinical practice, healthcare providers should tailor educational interventions and guidance specifically for pregnant women who are not medical professionals and those who have not received education and guidance related to constipation during pregnancy.

## Introduction

Functional constipation stands as a prevalent concern within the general population, assuming particular significance during pregnancy and the postpartum period (1). Constipation is commonly reported during pregnancy, ranking as the second most frequent complaint among pregnant women (2). This condition represents a frequent clinical syndrome with prevalence rates ranging from 2.6 to 24.8% in Asia and affecting up to 40% of pregnant women (2, 3). The impact of constipation extends beyond discomfort, reducing quality of life and increasing the burden on healthcare systems. Chronic constipation can lead to straining, which may injure the pudendal nerve, weaken the pelvic floor, or impair the supportive functions of the pelvic structures (4). Furthermore, if left untreated, chronic constipation can result in serious complications such as fecal impaction, incontinence, bowel damage, bleeding, hemorrhoids, and anal fissures (5). The hormone progesterone assumes a pivotal role during pregnancy, not only in relaxing smooth muscles in venous walls but also in the intestines, resulting in diminished motility and an increased risk of constipation (6). This risk escalates notably in late pregnancy due to decreased intestinal movement, substantial hormonal fluctuations, and delayed bowel emptying (7). Moreover, pregnant women exhibit heightened susceptibility to the effects of iron supplements and other prenatal vitamins, which can exacerbate constipation symptoms (8, 9). Despite being a common occurrence in pregnant women, constipation can adversely impact their quality of life and overall health. The discomfort associated with constipation can give rise to various related issues, including hemorrhoids, anal fissures, and rectal prolapse, thereby adversely affecting pregnant women’s quality of life, sleep, emotional well-being, and daily activities (10, 11). Addressing these concerns through targeted interventions is imperative for enhancing the well-being and health outcomes of pregnant women.

The Knowledge-Attitude-Practice (KAP) theory assumes a pivotal role in shaping human health behaviors (12). It is commonly employed alongside the KAP questionnaire to thoroughly gauge the knowledge, attitudes, and behaviors of the specific population within the healthcare sphere. Furthermore, it aids in evaluating the receptiveness and extent of acceptance of relevant information (13). This model, integral to health literacy, is underpinned by the fundamental premise that knowledge exerts a positive influence on attitudes, and these attitudes, in turn, shape individual practices (14). Given that constipation can induce discomfort in pregnant women, antenatal care during pregnancy assumes paramount importance for maternal and child health. Understanding and managing constipation issues during pregnancy are crucial for delivering high-quality prenatal care. Additionally, comprehending pregnant women’s attitudes and practices can help unveil potential issues and challenges, thereby contributing to the development of more effective intervention measures aimed at enhancing the quality of life and overall health of pregnant women. It is worth noting that there is currently a paucity of KAP studies in this area.

Shanghai, as the largest and most developed city in China, boasts a population of approximately 24 million people (15). Residents of this city tend to have relatively higher health awareness, which facilitates the dissemination and adoption of health-related information and practices. This heightened awareness among Shanghai’s populace can be instrumental in effectively promoting health education and interventions. Hence, this study aimed to investigate the KAP concerning constipation during pregnancy among pregnant women in Shanghai. The findings of this study may provide valuable insights for improving the management of constipation in pregnant women, potentially leading to better health outcomes and enhanced quality of life for this population.

## Materials and methods

### Study design and participants

This cross-sectional study was conducted at the Obstetrics and Gynaecology Hospital of Fudan University in Shanghai between August and October 2023. The inclusion criteria were as follows: (1) women over 18 years of age; and (2) pregnant women who attended antenatal examinations at our hospital during the study period. Participating pregnant women joined the hospital’s WeChat group via an outpatient QR code scan to complete the questionnaire. The exclusion criteria included: (1) questionnaire completion times less than 96 s or exceeding 1800 s; (2) cognitive impairments; and (3) refusal to participate in the study. The study received ethical approval from the Ethics Committee of the Obstetrics and Gynaecology Hospital of Fudan University, and informed consent was secured from all study participants.

### Procedures

The questionnaire was initially designed by the author and subsequently revised by two obstetrical experts to enhance its content accuracy. Following this initial design phase, a pilot survey was conducted using a small sample group to assess the questionnaire’s reliability. In the outpatient department, patients scanned a QR code to access and complete the electronic questionnaire. A total of 68 responses were collected during this pilot phase. The Cronbach’s α coefficient for internal consistency was determined to be 0.732, indicating conformity to established reliability standards. Consequently, no modifications were made to the questionnaire before its wider deployment in the study.

The final questionnaire, in Chinese, comprised four dimensions: (1) demographic information, consisting of 11 questions; (2) the knowledge dimension, including 6 questions and 26 sub-questions, with a scoring system assigning 1 point for correct answers and 0 points for unclear or incorrect responses, resulting in a score range of 0–26 points; (3) the attitude dimension, containing 8 questions, with only the 7th question being open-ended, and the remaining 7 questions scored on a 5-point Likert scale, ranging from 1 to 5 according to the degree of attitude, resulting in a score range of 7–35 points; and (4) the practice dimension, consisting of 17 questions, with questions 9, 10, and 16 not being scored. The rest of the questions employed a five-point Likert scale, with scores ranging from 1 to 5 based on the degree of practice, resulting in a score range of 14–70 points. Adequate knowledge, positive attitudes, and proactive practices were defined as achieving scores surpassing 70% of the maximum possible score in each respective section (16).

The sample size for our study was calculated using a standard statistical formula. With a confidence level of 95% (*z* = 1.96), an estimated proportion (p) of 0.5, and a margin of error (e) of 0.05, the formula *n* = *z*^2^ * *p* * (1 – *p*)/*e*^2^ yielded a sample size of approximately 384. We employed a convenience sampling method for this study. Before data collection, participants were thoroughly informed about the content and purpose of the study, assured of the non-invasive nature regarding their physical and mental health, and guaranteed confidentiality concerning their personal information. Only those who provided informed consent were included in the study. The questionnaire was administered using an online platform developed with the WeChat-based Questionnaire Star applet. A QR code was generated and distributed via WeChat to facilitate easy access and completion of the questionnaire. To maintain high data quality and ensure the completeness of the responses, the system was set to allow only one submission per IP address, and all response fields in the questionnaire were made mandatory. The research team conducted a meticulous review of all submissions to check for completeness, internal consistency, and logical coherence, thereby ensuring the reliability of the collected data.

### Statistical analyses

Descriptive analysis was performed for demographic data and Knowledge, Attitude, and Practice (KAP) scores of the participants. Continuous data were presented as median (IQR), while categorical data representing responses to various questions for different demographic characteristics were expressed as *n* (%). To compare differences in knowledge (K), attitude (A), and practice (P) scores among survey participants with different demographic characteristics, the following approaches were adopted: for continuous variables, a normality test was initially executed. If the data adhered to a normal distribution, a t-test was employed for comparisons between two groups. Conversely, if the data did not follow a normal distribution, a Mann–Whitney test was utilized for comparisons between two groups. For three or more groups with normally distributed data and equal variances, an ANOVA test was conducted. If the data did not meet the criteria for normality, a Kruskal-Wallis test was applied. Univariate logistic regression was undertaken with knowledge, attitude, and practice scores as dependent variables, using the median values of knowledge, attitude, and practice scores as the cut-off points. This analysis aimed to explore the influencing factors of knowledge, attitude, and practice scores. Multivariate logistic regression was employed using “Enter” method which is the default option on many statistical programs, all the input variables are entered simultaneously (17). All variables with a *p*-value less than 0.1 from the univariate analysis, such as education level, occupation type, and receipt of guidance, were included simultaneously in the model. This threshold (*p* < 0.10, instead of the usual *p* < 0.05) was chosen to balance the inclusion of potentially relevant variables while avoiding the introduction of excessive noise, as might occur with a higher threshold (18–20). This method is supported by literature that discusses the benefits of a more inclusive threshold in exploratory studies to ensure important variables are not excluded prematurely (18, 21). Statistical significance was considered at *p* < 0.05. The statistical analysis was performed using SPSS 26.0 (IBM Corp., Armonk, NY, USA).

## Results

In this study, a total of 517 questionnaires were collected, with 241 (46.6%) participants falling within the age bracket of 30–34 years, 311 (60.2%) attaining education up to the level of a Bachelor’s degree or college, and 349 (67.5%) being employed in white-collar or company positions. Furthermore, 349 (67.5%) were nulliparas, and the majority experienced singleton pregnancies (97.3%). A total of 358 (69.2%) reported experiencing constipation during pregnancy, while 236 (45.6%) reported constipation before pregnancy, with 116 (49.2%) indicating a duration of less than 1 year. A significant proportion, 380 (73.5%), had not received education and guidance about constipation during pregnancy. The median (25th percentile, 75th percentile) scores for knowledge (possible range: 0–26), attitude (possible range: 7–35), and practice (possible range: 14–70) were 22 (18, 24), 26 (23, 29), and 51 (46, 56), respectively. The knowledge score exhibited variation among pregnant women based on different occupation types (*p* = 0.005) and whether they had received education and guidance related to constipation during pregnancy (*p* < 0.001). Regarding attitude scores, differences were noted among pregnant women with varying gestational weeks (*p* = 0.021), singleton/twin pregnancies (*p* = 0.041), those who experienced constipation during pregnancy (*p* < 0.001), and those who experienced constipation before pregnancy (*p* = 0.004). Practice scores differed among pregnant women based on age (*p* < 0.001), education (*p* = 0.031), occupation type (*p* = 0.015), whether they experienced constipation during pregnancy (*p* = 0.027), whether they experienced constipation before pregnancy (*p* < 0.001), and whether they received education and guidance related to constipation during pregnancy (*p* = 0.016) (Table 1).

**Table 1:** Baseline sheet.

Variables	*N* (%)	Knowledge (K)		Attitude (A)		Practice (P)	
		Median (25% quartile, 75% quartile)	*p*-value	Median (25% quartile, 75% quartile)	*p*-value	Median (25% quartile, 75% quartile)	*p*-value
Total	517	22 (18, 24)		26 (23, 29)		51 (46, 56)	
Age			0.097		0.156		**<0.001**
Under 20 years old	1 (0.2)	22 (22, 22)		35 (35, 35)		32 (32, 32)	
20–24 years old	11 (2.1)	23 (22, 25)		24 (22, 28)		42 (38, 52)	
25–29 years old	133 (25.7)	22 (18, 24)		26 (22, 28)		48 (44, 54)	
30–34 years old	241 (46.6)	21 (18, 24)		26 (23, 29)		52 (47, 57)	
35–39 years old	106 (20.5)	22 (20, 24)		27 (23, 30)		53 (48, 57)	
40 years old and above	25 (4.8)	20 (17, 22)		25 (23, 28)		51 (48, 55)	
Education			0.092		0.332		**0.031**
Middle school	20 (3.9)	19 (15, 24)		26.5 (24.5, 29.5)		46.5 (42.5, 53.5)	
High School/Vocational High School	24 (4.6)	20 (14.5, 24)		25 (23, 28.5)		52 (45.5, 55.5)	
Undergraduate/Junior college	311 (60.2)	21 (18, 24)		27 (23, 29)		51 (45, 56)	
Postgraduate and above	162 (31.3)	22 (19, 24)		26 (22, 29)		52 (48, 57)	
Occupation type			**0.005**		0.967		**0.015**
White-collar/company employee	349 (67.5)	22 (18, 24)		26 (23, 29)		51 (46, 56)	
Education personnel	54 (10.4)	21 (17, 24)		26.5 (22, 29)		54 (49, 58)	
Medical personnel	36 (7.0)	24 (21, 25)		26 (22, 29)		51.5 (45.5, 58)	
Outdoor worker	1 (0.2)	20 (20, 20)		25 (25, 25)		38 (38, 38)	
Labor-intensive work	9 (1.7)	19 (15, 24)		25 (24, 28)		47 (42, 52)	
Other	68 (13.2)	20 (17, 24)		26 (23, 29)		50.5 (45.5, 55)	
Weeks of pregnancy			0.655		**0.021**		0.878
≤12 weeks	64 (12.4)	21.5 (18.5, 24)		27 (25, 29.5)		50 (46, 55)	
13–20 weeks	124 (24.0)	22 (18, 24)		27 (24, 29)		52 (46, 55)	
21–28 weeks	99 (19.1)	22 (19, 24)		27 (24, 30)		51 (46, 57)	
29–36 weeks	115 (22.2)	21 (18, 24)		26 (22, 29)		51 (46, 57)	
≥37 weeks	115 (22.2)	22 (18, 24)		25 (22, 28)		51 (46, 57)	
First pregnancy			0.484		0.964		0.874
Yes	349 (67.5)	22 (18, 24)		26 (23, 29)		51 (46, 56)	
No	168 (32.5)	21 (17.5, 24)		26 (23, 29)		51.5 (46, 56)	
Singleton or a twin			0.487		**0.041**		0.263
Singleton	503 (97.3)	22 (18, 24)		26 (23, 29)		51 (46, 56)	
Twin	14 (2.7)	22.5 (18, 25)		29 (26, 31)		54 (50, 57)	
Constipation in pregnancy			0.239		**<0.001**		**0.027**
Yes	358 (69.2)	21 (18, 24)		27 (24, 30)		51 (46, 56)	
No	159 (30.8)	22 (18, 24)		25 (22, 28)		52 (47, 57)	
Constipation before pregnancy			0.189		**0.004**		**<0.001**
Yes	236 (45.6)	21 (18, 24)		27 (24, 30)		49.5 (45, 54.5)	
No	281 (54.4)	22 (19, 24)		26 (22, 29)		53 (47, 57)	
Duration of constipation (*n* = 236)			0.211		0.054		0.423
Less than 1 year	116 (49.2)	20 (18, 24)		26.5 (23.5, 29)		49.5 (45, 55)	
1–3 years	41 (17.4)	21 (18, 24)		26 (24, 29)		51 (45, 55)	
More than 3 years	79 (33.5)	22 (19, 24)		28 (25, 31)		49 (44, 54)	
History of smoking or alcohol consumption			0.149		0.345		0.434
No	501 (96.9)	22 (18, 24)		26 (23, 29)		51 (46, 56)	
History of smoking	10 (1.9)	21.5 (17, 25)		22.5 (19, 30)		53.5 (50, 55)	
History of alcohol consumption	2 (0.4)	16.5 (15, 18)		25.5 (24, 27)		49 (45, 53)	
History of both smoking and alcohol consumption	4 (0.8)	19 (15, 20)		25 (22.5, 27.5)		45 (42.5, 50)	
Receiving education and guidance related to constipation in pregnancy			**<0.001**		0.078		**0.016**
Yes	137 (26.5)	23 (20, 25)		26 (22, 28)		52 (47, 57)	
No	380 (73.5)	21 (18, 24)		27 (23, 29)		51 (46, 55.5)	

The bold values indicate that the *P*-value was less than 0.05, which signified statistical significance.

In the knowledge dimension, 79.3% of pregnant women exhibit an awareness of constipation during pregnancy (K1). Regarding potential symptoms of constipation in pregnancy, awareness exceeds 70% for all of them (K2). In terms of factors that may contribute to constipation during pregnancy, awareness surpasses 85% for all, except for the association with smoking and drinking (K3f), which only 54.0% are cognizant of. Notably, only 48.5% are aware that constipation during pregnancy may lead to difficult childbirth (K4d). In the realm of measures for preventing and treating constipation during pregnancy, the least-known is the consumption of legumes (red beans, green beans, etc.) (K5d, only 55.7%). Surprisingly, 90.7% recognize that establishing a habit of regular bowel movements can prevent constipation (K6) (Supplementary Table S1).

Regarding attitudes toward constipation during pregnancy, 42.7% of respondents perceive it as very common (A1). The terms “very common” and “less common” reflect the personal perspectives of the respondents based on their understanding and experience. These terms were used to capture the subjective views of participants. Furthermore, a notable portion of participants express various concerns related to this issue. Specifically, 35.6% are apprehensive about experiencing constipation during pregnancy (A2), while 40.4% indicate that they would feel distressed and troubled if they were to encounter it (A4). Additionally, 27.1% express concerns about how constipation might impact the late-term health of the fetus (A5). Moreover, 28.0% are uncertain about whether they are concerned about the potential risk of miscarriage or premature birth in relation to constipation during pregnancy (A4). 46.2% of respondents emphasize the importance of preventive measures, believing they can have some effect in averting constipation during pregnancy (A6). 60.3% of participants favor the use of lactulose to address constipation during pregnancy (A7). Furthermore, 59.4% variably agree that regulating their emotions can have a positive impact on alleviating constipation in pregnancy (A8) (Supplementary Table S2).

The practices of pregnant women exhibited variability. 30.9% predominantly adopted a sedentary lifestyle or rested in bed during pregnancy, whereas 30.6% engaged in appropriate daily exercise (P1). Furthermore, 31.9% occasionally stayed up late (P2), 38.9% occasionally consumed spicy and stimulating foods (P3), and sweets (P4). Additionally, 41.6% reported having three regular meals (P11), 47.0% included staple food in every meal (P6), and 72.1% regularly consumed green vegetables (P7). Among those with a preference for meat, red meat was the most popular (P10.1, 75.3%). Among those with a preference for vegetarian food, green leafy vegetables were the most popular (P10.2, 88.1%). Additionally, 57.1% paid regular attention to their bowel situation (P12). When experiencing constipation during pregnancy, 42.9% chose to consult a hospital for treatment, followed by 34.4% consulting a pharmacy for treatment (P15). 74.3% stated that they usually or always take the initiative to regulate their emotions (P16) (Supplementary Table S3).

Correlation analysis revealed that knowledge was positively correlated with attitude (*r* = 0.150, *p* < 0.001) and practice (*r* = 0.039, *p* = 0.381). Conversely, attitude exhibited a negative correlation with practice (*r* = −0.049, *p* = 0.268) (Supplementary Table S4).

In the multivariate analysis, variables with *p*-values less than 0.1 from the univariate analysis were incorporated. The results revealed that being medical personnel (OR = 2.222, 95% CI: [1.026–4.815], *p* = 0.043) and receiving education and guidance related to constipation during pregnancy (OR = 0.432, 95% CI: [0.285–0.654], *p* < 0.001) were significantly associated with knowledge (Table 2). Meanwhile, being aged 30–34 years old (OR = 1.571, 95% CI: [1.021–2.418], *p* = 0.040) was significantly associated with a positive attitude. Conversely, being in the gestational period of 29–36 weeks (OR = 0.602, 95% CI: [0.370–0.979], *p* = 0.041), having a gestational period greater than or equal to 37 weeks (OR = 0.451, 95% CI: [0.278–0.734], *p* = 0.001), and not experiencing constipation during pregnancy (OR = 0.553, 95% CI: [0.366–0.837], *p* = 0.005) were significantly associated with a negative attitude (Table 3). Furthermore, being aged 30–34 years old (OR = 2.745, 95% CI: [1.741–4.327], *p* < 0.001), being aged 35 years old and above (OR = 2.514, 95% CI: [1.511–4.182], *p* < 0.001), being in an educational profession (OR = 2.310, 95% CI: [1.204–4.433], *p* = 0.012), and not experiencing constipation before pregnancy (OR = 1.894, 95% CI: [1.285–2.792], *p* = 0.001) were significantly associated with proactive practice (Table 4).

**Table 2:** Univariate and multivariate regression analysis (Knowledge dimension).

Cut-off value: ≥22/<22	No.	Univariate		Multivariate (regression method = input)	
		OR (95%CI)	*p*-value	OR (95%CI)	*p*-value
**Age**					
29 years old and below	80/145	ref.			
30–34 years old	115/241	0.742 (0.490, 1.121)	0.156		
35 years old and above	68/131	0.877 (0.546, 1.409)	0.587		
**Education**					
High School/Vocational High School and below	18/44	0.514 (0.261, 1.011)	0.054	0.568 (0.250, 1.289)	0.176
Undergraduate/Junior college	152/311	0.709 (0.484, 1.040)	0.078	0.704 (0.474, 1.047)	0.083
Postgraduate and above	93/162	ref.		ref.	
**Occupation type**					
White-collar/company employee	181/349	ref.		ref.	
Education personnel	24/54	0.743 (0.417, 1.321)	0.311	0.690 (0.382, 1.248)	0.220
Medical personnel	26/36	2.413 (1.130, 5.155)	**0.023**	2.222 (1.026, 4.815)	**0.043**
Outdoor worker	0/1	/	/	/	/
Labor-intensive work	4/9	0.743 (0.196, 2.812)	0.661	0.987 (0.235, 4.148)	0.986
Other	28/68	0.650 (0.384, 1.100)	0.109	0.822 (0.446, 1.515)	0.531
**Weeks of pregnancy**					
≤20 weeks	97/188	ref.			
21 ~ 28 weeks	53/99	1.081 (0.664, 1.760)	0.755		
29 ~ 36 weeks	55/115	0.860 (0.540, 1.368)	0.524		
≥37 weeks	58/115	0.955 (0.600, 1.518)	0.844		
**First pregnancy**					
Yes	184/349	ref.			
No	79/168	0.796 (0.551, 1.151)	0.225		
**Singleton or a twin**					
Singleton	255/503	ref.			
Twin	8/14	1.297 (0.444, 3.791)	0.635		
**Constipation in pregnancy**					
Yes	176/358	ref.			
No	87/159	1.250 (0.859, 1.818)	0.244		
**Constipation before pregnancy**					
Yes	113/236	ref.			
No	150/281	1.246 (0.881, 1.763)	0.213		
**History of smoking or alcohol consumption**					
No	258/501	ref.			
Yes	5/16	0.428 (0.147, 1.250)	0.121		
**Receiving education and guidance related to constipation in pregnancy**					
Yes	91/137	ref.		ref.	
No	172/380	0.418 (0.278, 0.629)	***p* < 0.001**	0.432 (0.285, 0.654)	***p* < 0.001**

The bold values indicate that the *P*-value was less than 0.05, which signified statistical significance.

**Table 3:** Univariate and multivariate regression analysis (Attitude dimension).

Cut-off value: ≥26/<26	No.	Univariate		Multivariate (regression method = input)	
		OR (95%CI)	*p*-value	OR (95%CI)	*p*-value
**Age**					
29 years old and below	72/145	ref.		ref.	
30–34 years old	147/241	1.586 (1.046, 2.403)	**0.030**	1.571 (1.021, 2.418)	**0.040**
35 years old and above	71/131	1.200 (0.747, 1.926)	0.451	1.234 (0.756, 2.015)	0.399
**Education**					
High School/Vocational High School and below	24/44	1.142 (0.585, 2.229)	0.697		
Undergraduate/Junior college	183/311	1.361 (0.929, 1.994)	0.114		
Postgraduate and above	83/162	ref.			
**Occupation type**					
White-collar/company employee	198/349	ref.			
Education personnel	32/54	1.109 (0.619, 1.986)	0.727		
Medical personnel	18/36	0.763 (0.384, 1.516)	0.439		
Outdoor worker	0/1	/	/		
Labor-intensive work	4/9	0.610 (0.161, 2.311)	0.467		
Other	38/68	0.966 (0.572, 1.630)	0.897		
**Weeks of pregnancy**					
≤20 weeks	117/188	ref.		ref.	
21 ~ 28 weeks	61/99	0.974 (0.590, 1.608)	0.918	0.892 (0.534, 1.491)	0.663
29 ~ 36 weeks	60/115	0.662 (0.414, 1.059)	0.085	0.602 (0.370, 0.979)	**0.041**
≥37 weeks	52/115	0.501 (0.313, 0.802)	**0.004**	0.451 (0.278, 0.734)	**0.001**
**First pregnancy**					
Yes	197/349	ref.			
No	93/168	0.957 (0.661, 1.386)	0.815		
**Singleton or a twin**					
Singleton	279/503	ref.			
Twin	11/14	2.944 (0.811, 10.680)	0.101		
**Constipation in pregnancy**					
Yes	219/358	ref.		ref.	
No	71/159	0.512 (0.351, 0.747)	**0.001**	0.553 (0.366, 0.837)	**0.005**
**Constipation before pregnancy**					
Yes	147/236	ref.		ref.	
No	143/281	0.627 (0.441, 0.892)	**0.009**	0.781 (0.532, 1.146)	0.206
**History of smoking or alcohol consumption**					
No	284/501	ref.			
Yes	6/16	0.458 (0.164, 1.281)	0.137		
**Receiving education and guidance related to constipation in pregnancy**					
Yes	73/137	ref.			
No	217/380	1.167 (0.788, 1.728)	0.440		

The bold values indicate that the *P*-value was less than 0.05, which signified statistical significance.

**Table 4:** Univariate and multivariate regression analysis (Practice dimension).

Cut-off value: ≥51/<51	No.	Univariate		Multivariate (regression method = input)	
		OR (95%CI)	*p*-value	OR (95%CI)	*p*-value
**Age**					
29 years old and below	55/145	ref.		ref.	
30–34 years old	145/241	2.472 (1.619, 3.773)	***p* < 0.001**	2.745 (1.741, 4.327)	***p* < 0.001**
35 years old and above	77/131	2.333 (1.439, 3.783)	**0.001**	2.514 (1.511, 4.182)	***p* < 0.001**
**Education**					
High School/Vocational High School and below	23/44	0.734 (0.376, 1.434)	0.365	1.069 (0.464, 2.464)	0.875
Undergraduate/Junior college	157/311	0.683 (0.465, 1.004)	0.052	0.800 (0.531, 1.203)	0.283
Postgraduate and above	97/162	ref.		ref.	
**Occupation type**					
White-collar/company employee	182/349	ref.		ref.	
Education personnel	38/54	2.179 (1.171, 4.054)	**0.014**	2.310 (1.204, 4.433)	**0.012**
Medical personnel	19/36	1.026 (0.516, 2.039)	0.943	1.032 (0.503, 2.116)	0.932
Outdoor worker	0/1	/	/	/	/
Labor-intensive work	4/9	0.734 (0.194, 2.780)	0.649	0.998 (0.233, 4.271)	0.998
Other	34/68	0.918 (0.546, 1.543)	0.746	0.954 (0.513, 1.774)	0.882
**Weeks of pregnancy**					
≤20 weeks	101/188	ref.			
21 ~ 28 weeks	52/99	0.953 (0.585, 1.552)	0.847		
29 ~ 36 weeks	62/115	1.008 (0.633, 1.605)	0.974		
≥37 weeks	62/115	1.008 (0.633, 1.605)	0.974		
**First pregnancy**					
Yes	187/349	ref.			
No	90/168	1.000 (0.691, 1.446)	0.998		
**Singleton or a twin**					
Singleton	267/503	ref.			
Twin	10/14	2.210 (0.684, 7.139)	0.185		
**Constipation in pregnancy**					
Yes	180/358	ref.		ref.	
No	97/159	1.547 (1.058, 2.263)	**0.024**	1.450 (0.947, 2.221)	0.087
**Constipation before pregnancy**					
Yes	105/236	ref.		ref.	
No	172/281	1.969 (1.385, 2.798)	***p* < 0.001**	1.894 (1.285, 2.792)	**0.001**
**History of smoking or alcohol consumption**					
No	268/501	ref.			
Yes	9/16	1.118 (0.410, 3.048)	0.828		
**Receiving education and guidance related to constipation in pregnancy**					
Yes	81/137	ref.			
No	196/380	0.736 (0.496, 1.094)	0.130		

The bold values indicate that the *P*-value was less than 0.05, which signified statistical significance.

## Discussion

Pregnant women exhibited a satisfactory level of knowledge, positive attitudes, and proactive practices toward constipation during pregnancy. To improve clinical practice, it is recommended to target educational efforts toward those lacking prior knowledge, customize interventions for specific age groups, and promote preventive practices among those without a history of constipation before pregnancy.

The pregnant women in this study demonstrated a generally commendable level of knowledge, positive attitudes, and proactive practices concerning constipation during pregnancy, as reflected by median scores within acceptable ranges. The observed variations in knowledge, attitudes, and practices based on demographic factors and prior education are noteworthy. Firstly, it is encouraging to observe that being a medical professional and receiving education on constipation during pregnancy were independently associated with higher knowledge scores. This suggests that healthcare providers can play a crucial role in disseminating accurate information to pregnant women, potentially by integrating essential education into routine antenatal care (22, 23). Nevertheless, despite this generally positive trend, certain groups displayed a less favorable attitude, notably pregnant women in later stages of pregnancy and those who have experienced constipation during pregnancy or before. These findings underline the importance of early interventions and education to foster more positive attitudes, potentially reducing the discomfort and complications associated with constipation in the later stages of pregnancy (24, 25). It is evident that tailored educational programs and support mechanisms for these specific groups can contribute to enhanced clinical outcomes. Furthermore, the factors independently associated with proactive practices, such as age, occupation, and pre-pregnancy constipation history, suggest that specific subpopulations may be more inclined to adopt recommended practices. This insight can guide clinicians in identifying those at greater risk and offer personalized guidance to improve proactive practices among pregnant women, ultimately leading to better management of constipation during pregnancy (26).

The findings within the knowledge dimension of this investigation reveal a generally positive comprehension among gravid females concerning constipation during gestation. It is imperative, however, to concentrate on rectifying specific deficiencies and proffering initiatives to augment clinical proficiency in the management of this physiological state. Notably, the preponderance of participants adeptly identified the symptoms and ramifications of constipation during pregnancy, indicating a robust foundational knowledge. Nevertheless, a smaller subset accurately recognized the measures for averting or ameliorating constipation, with certain participants expressing ambiguity regarding recommended practices. This incongruity accentuates an arena where clinical efficacy can be ameliorated. To bridge this lacuna, healthcare providers should accord precedence to patient education and counseling concerning preventative measures, such as dietary recommendations, hydration, and regular bowel habits (27). Furthermore, exertions to augment knowledge and awareness should be continual and bespoke to rectify particular misconceptions or uncertainties uncovered in this inquiry, guaranteeing that all gravid females receive comprehensive guidance and support for the management of constipation during gestation (28–30). A targeted educational approach, particularly centered on preventive strategies, has the potential to yield superior clinical outcomes, heightened patient contentment, and overall well-being during pregnancy (31, 32).

The findings derived from the attitude dimension of this investigation yield valuable insights into the perceptions and concerns of gravid females regarding constipation during gestation, illuminating avenues for refining clinical practice. Evidently, a substantial proportion of participants acknowledge constipation during pregnancy as a common occurrence; however, there exists a spectrum of sentiments encompassing worry, distress, and annoyance associated with this physiological state. Some articulate apprehensions regarding potential repercussions on pregnancy and fetal health, underscoring the necessity for proactive management. Notably, the majority of participants appreciate the significance of preventive measures through dietary and lifestyle modifications in addressing constipation during pregnancy. Nevertheless, there exists room for enhancement in elevating awareness about specific intervention options such as lactulose, probiotics, and laxatives, alongside mood regulation to alleviate constipation symptoms. In consideration of these outcomes, refining clinical practice involves the provision of personalized counseling and support to assuage concerns and anxieties, particularly for those experiencing heightened distress (33, 34). Furthermore, an opportunity arises to enlighten gravid females on diverse management options, underscoring the efficacy of dietary and lifestyle adjustments, and enhancing awareness about specific interventions when warranted (28, 35). An integrated approach that addresses the psychological and physiological dimensions of constipation during gestation can contribute to superior clinical outcomes and patient well-being.

The practice dimension of this investigation unveils pivotal insights into the behaviors and habits exhibited by gravid females in Shanghai with regard to constipation during gestation. It is apparent that various lifestyle factors, such as frequent sitting or lying down, late-night wakefulness, and dietary preferences, are prevalent among the participants, potentially conferring to constipation issues. Furthermore, the findings disclose that certain pregnant women adopt unhealthy eating practices, including the consumption of spicy or sweet foods and substituting water with less hydrating beverages. Additionally, there exists a deficiency in the emphasis on regular exercise and the inclusion of green vegetables in the diet. Of particular note, a considerable number of participants neglect monitoring their bowel movements, surpassing the recommended duration for such activities, and exhibit a tendency to postpone bowel movements upon feeling the urge. Rectifying these shortcomings in clinical practice necessitates the provision of comprehensive education and counseling regarding the significance of regular exercise, maintenance of a balanced diet with a focus on high-fiber foods and adequate hydration, and the cultivation of healthy bowel habits (36, 37). Initiatives targeted at heightening awareness and effecting behavioral changes in these domains can substantially contribute to the prevention and management of constipation during gestation, ultimately augmenting the overall well-being of expectant mothers.

Given the observed gaps in KAP concerning constipation during pregnancy as indicated in the study, it becomes imperative to implement tailored educational initiatives that address specific needs. Tailored education should encompass detailed discussions, visual aids on symptoms and potential outcomes, and integrated information sessions during routine antenatal care. These could include live demonstrations on dietary modifications, physical activity suitable for pregnancy, and stress management techniques such as prenatal yoga. Furthermore, personalized counseling and support groups can offer individual guidance and community learning opportunities. Regular assessments during prenatal visits would also enable healthcare providers to continuously adapt educational content, ensuring it meets the dynamic needs of pregnant women.

The correlation analysis carried out in this study has unveiled associations between knowledge, attitude, and practice regarding constipation during pregnancy. These findings underscore the interplay among these factors, wherein knowledge exhibits a positive correlation with attitude, and attitude, in turn, demonstrates a negative correlation with practice. These results intimate that while augmenting knowledge can cultivate more positive attitudes, there exists a divergence between attitudes and tangible practices. For the enhancement of clinical practice, interventions should concentrate on addressing this disparity and equipping gravid females with the requisite tools and motivation to translate their positive attitudes into proactive behaviors (38, 39).

Several limitations merit consideration when interpreting the results of this study. Firstly, the study employed a self-administered questionnaire, which may be susceptible to response bias and self-reporting inaccuracies due to an inadequate provision for precise quantification. Secondly, the cross-sectional design of the study restricts our capacity to establish causal relationships between variables. Thirdly, the study was conducted at the Obstetrics and Gynaecology Hospital of Fudan University in Shanghai, where the majority of individuals possess a high level of education and income. The dietary structure reflects certain characteristics of metropolitan areas, and the findings may not be readily generalizable to other regions or populations. Additionally, the study did not delve into other potential factors influencing knowledge, attitudes, and practices, such as cultural or socioeconomic factors.

## Conclusion

Pregnant women demonstrated sufficient knowledge, a positive attitude, and proactive practices toward constipation during pregnancy. In light of our findings, we propose that healthcare providers in Shanghai deliver tailored education and guidance on pregnancy-related constipation, especially targeting those without a medical background. Furthermore, there should be an emphasis on fostering positive attitudes and proactive practices, particularly in pregnant women aged 30–34 and those aged 35 years and above. Lastly, there is a need to promote preventive practices among women with no prior history of constipation before pregnancy.

## Data availability statement

The original contributions presented in the study are included in the article/Supplementary material, further inquiries can be directed to the corresponding author.

## Ethics statement

The studies involving humans were approved by the Medical Ethics Committee of Obstetrics and Gynecology Hospital of Fudan University (2023-91). The studies were conducted in accordance with the local legislation and institutional requirements. Written informed consent for participation was not required from the participants or the participants’ legal guardians/next of kin in accordance with the national legislation and institutional requirements.

## Author contributions

LL: Formal analysis, Writing – original draft. YY: Investigation, Writing – original draft. WG: Writing – review and editing. RH: Writing – review and editing. HZ: Investigation, Formal analysis, Writing – review and editing.

## Supplementary material

SUPPLEMENTARY FIGURE S1 Percentile graph of knowledge dimension distribution.
