# Supplementary figures and images for: Knowledge, attitude and practice regarding constipation in pregnancy among pregnant women in Shanghai: a cross-sectional study
Source: Front Public Health. 2024 Jul 18;12:1378301. doi: 10.3389/fpubh.2024.1378301 (PMC11291461; doi:10.3389/fpubh.2024.1378301)

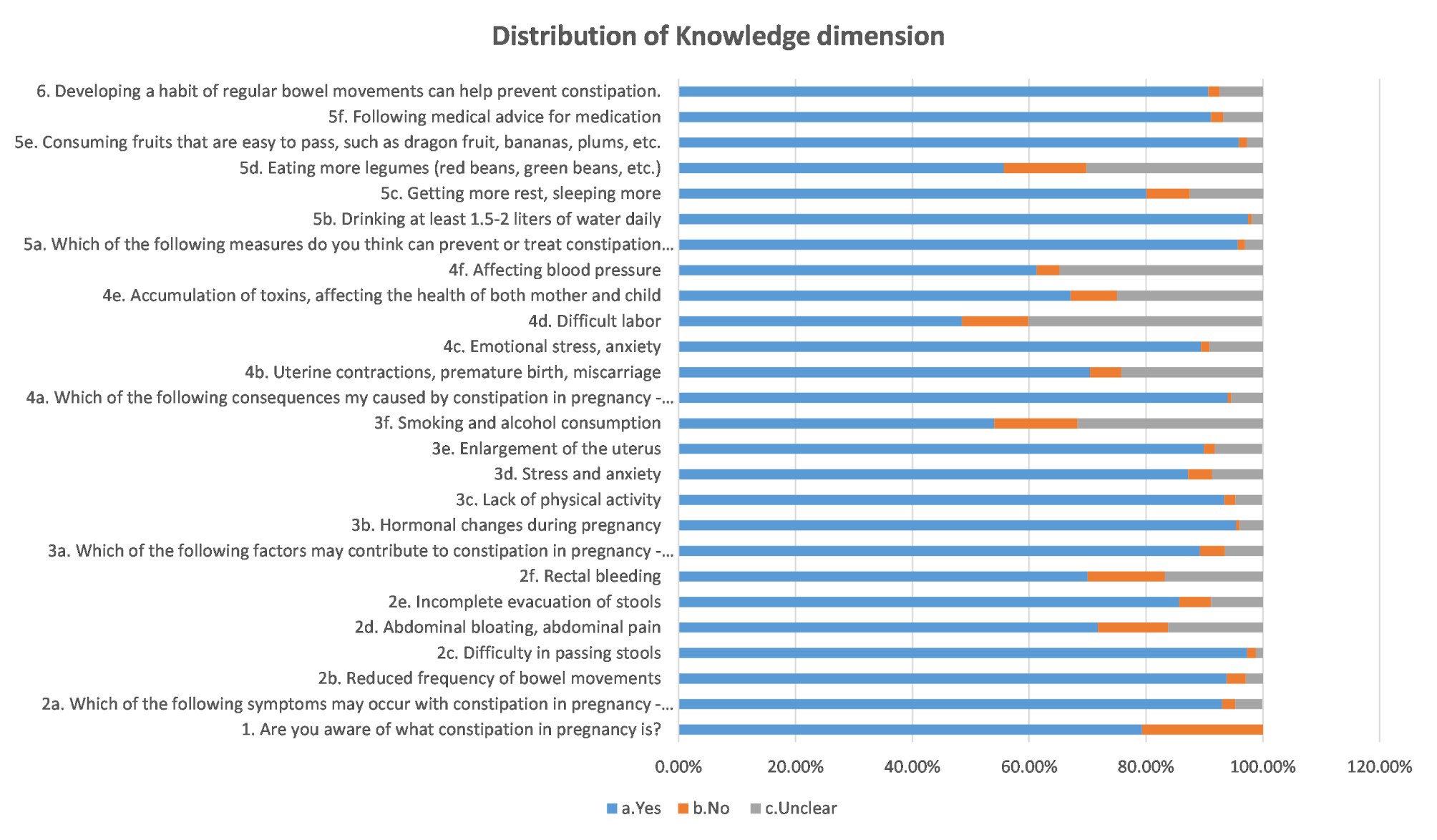

Supplement: SUPPLEMENTARY FIGURE S1 — Percentile graph of knowledge dimension distribution. [file Image_1.TIFF]
