# Supplementary material for: Knowledge, attitude and practice regarding constipation in pregnancy among pregnant women in Shanghai: a cross-sectional study
Source: Front Public Health. 2024 Jul 18;12:1378301. doi: 10.3389/fpubh.2024.1378301 (PMC11291461; doi:10.3389/fpubh.2024.1378301)
Supplement: Supplementary file 2 [file Table_1.DOCX]

**Supplementary table 1 Distribution of Knowledge dimension**

|  | **a.Know (1)** | **b.Don't know (0)** | **c.Unsure (0)** |
| --- | --- | --- | --- |
| **1. Are you aware of what constipation in pregnancy is?** | **410(79.3)** | 107(20.7) | 0(0.0) |
|  | **a.Correct (1)** | **b.Wrong (0)** | **c.Unclear (0)** |
| **2a. Which of the following symptoms may occur with constipation in pregnancy - dry, hard stools** | **481(93.0)** | 12(2.3) | 24(4.6) |
|  | **a.Correct (1)** | **b.Wrong (0)** | **c.Unclear (0)** |
| **2b. Reduced frequency of bowel movements** | **485(93.8)** | 17(3.3) | 15(2.9) |
|  | **a.Correct (1)** | **b.Wrong (0)** | **c.Unclear (0)** |
| **2c. Difficulty in passing stools** | **503(97.3)** | 8(1.5) | 6(1.2) |
|  | **a.Correct (1)** | **b.Wrong (0)** | **c.Unclear (0)** |
| **2d. Abdominal bloating, abdominal pain** | **371(71.8)** | 62(12.0) | 84(16.2) |
|  | **a.Correct (1)** | **b.Wrong (0)** | **c.Unclear (0)** |
| **2e. Incomplete evacuation of stools** | **443(85.7)** | 28(5.4) | 46(8.9) |
|  | **a.Correct (1)** | **b.Wrong (0)** | **c.Unclear (0)** |
| **2f. Rectal bleeding** | **362(70.0)** | 68(13.2) | 87(16.8) |
|  | **a.Correct (1)** | **b.Wrong (0)** | **c.Unclear (0)** |
| **3a. Which of the following factors may contribute to constipation in pregnancy - changes in dietary habits** | **461(89.2)** | 22(4.3) | 34(6.6) |
|  | **a.Correct (1)** | **b.Wrong (0)** | **c.Unclear (0)** |
| **3b. Hormonal changes during pregnancy** | **493(95.4)** | 3(0.6) | 21(4.1) |
|  | **a.Correct (1)** | **b.Wrong (0)** | **c.Unclear (0)** |
| **3c. Lack of physical activity** | **483(93.4)** | 10(1.9) | 24(4.6) |
|  | **a.Correct (1)** | **b.Wrong (0)** | **c.Unclear (0)** |
| **3d. Stress and anxiety** | **451(87.2)** | 21(4.1) | 45(8.7) |
|  | **a.Correct (1)** | **b.Wrong (0)** | **c.Unclear (0)** |
| **3e. Enlargement of the uterus** | **465(89.9)** | 10(1.9) | 42(8.1) |
|  | **a.Correct (1)** | **b.Wrong (0)** | **c.Unclear (0)** |
| **3f. Smoking and alcohol consumption** | **279(54.0)** | 74(14.3) | 164(31.7) |
|  | **a.Correct (1)** | **b.Wrong (0)** | **c.Unclear (0)** |
| **4a. Which of the following consequences my caused by constipation in pregnancy - the development of hemorrhoids** | **486(94.0)** | 3(0.6) | 28(5.4) |
|  | **a.Correct (1)** | **b.Wrong (0)** | **c.Unclear (0)** |
| **4b. Uterine contractions, premature birth, miscarriage** | **364(70.4)** | 28(5.4) | 125(24.2) |
|  | **a.Correct (1)** | **b.Wrong (0)** | **c.Unclear (0)** |
| **4c. Emotional stress, anxiety** | **462(89.4)** | 8(1.5) | 47(9.1) |
|  | **a.Correct (1)** | **b.Wrong (0)** | **c.Unclear (0)** |
| **4d. Difficult labor** | **251(48.5)** | 59(11.4) | 207(40.0) |
|  | **a.Correct (1)** | **b.Wrong (0)** | **c.Unclear (0)** |
| **4e. Accumulation of toxins, affecting the health of both mother and child** | **347(67.1)** | 41(7.9) | 129(25.0) |
|  | **a.Correct (1)** | **b.Wrong (0)** | **c.Unclear (0)** |
| **4f. Affecting blood pressure** | **317(61.3)** | 20(3.9) | 180(34.8) |
|  | **a.Correct (1)** | **b.Wrong (0)** | **c.Unclear (0)** |
| **5a. Which of the following measures do you think can prevent or treat constipation in pregnancy - eating more high-fiber foods (such as enoki mushrooms, celery, chives, etc.)** | **495(95.7)** | 6(1.2) | 16(3.1) |
|  | **a.Correct (1)** | **b.Wrong (0)** | **c.Unclear (0)** |
| **5b. Drinking at least 1.5-2 liters of water daily** | **504(97.5)** | 3(0.6) | 10(1.9) |
|  | **a.Correct (0)** | **b.Wrong (1)** | **c.Unclear (0)** |
| **5c. Getting more rest, sleeping more** | **414(80.1)** | 38(7.4) | 65(12.6) |
|  | **a.Correct (1)** | **b.Wrong (0)** | **c.Unclear (0)** |
| **5d. Eating more legumes (red beans, green beans, etc.)** | **288(55.7)** | 73(14.1) | 156(30.2) |
|  | **a.Correct (1)** | **b.Wrong (0)** | **c.Unclear (0)** |
| **5e. Consuming fruits that are easy to pass, such as dragon fruit, bananas, plums, etc.** | **496(95.9)** | 7(1.4) | 14(2.7) |
|  | **a.Correct (1)** | **b.Wrong (0)** | **c.Unclear (0)** |
| **5f. Following medical advice for medication** | **471(91.1)** | 11(2.1) | 35(6.8) |
|  | **a.Yes (1)** | **b.No(0)** | **c.Unclear (0)** |
| **6. Developing a habit of regular bowel movements can help prevent constipation.** | **469(90.7)** | 10(1.9) | 38(7.4) |

**Supplementary table2 Distribution of Attitude dimension**

|  | **a. Uncommon (1)** | **b. Less common (2)** | **c. Unsure (3)** | **d. More common (4)** | **e.Very common (5)** |
| --- | --- | --- | --- | --- | --- |
| **1. Do you consider constipation in pregnancy to be very common?** | 4(0.8) | 5(1.0) | 92(17.8) | 195(37.7) | **221(42.7)** |
|  | **a. Not worried at all (1)** | **b. Not too worried (2)** | **c. Unsure (3)** | **d. More worried (4)** | **e. Very worried (5)** |
| **2. Are you worried about experiencing constipation in pregnancy?** | 47(9.1) | 63(12.2) | 82(15.9) | **184(35.6)** | 141(27.3) |
|  | **a.Not distressed and annoyed at all (1)** | **b.Not too distressed and annoyed (2)** | **c. Unsure (3)** | **d. More distressed and annoyed (4)** | **e. Very distressed and annoyed (5)** |
| **3. 3. Do you think constipation during pregnancy is distressing and annoying?** | 31(6.0) | 54(10.4) | 72(13.9) | **209(40.4)** | 151(29.2) |
|  | **a. Not worried at all (1)** | **b. Not too worried (2)** | **c. Unsure (3)** | **d. More worried (4)** | **e. Very worried (5)** |
| **4. Are you concerned that constipation in pregnancy may lead to a miscarriage or premature birth?** | 68(13.2) | 72(13.9) | **145(28.0)** | 132(25.5) | 100(19.3) |
|  | **a. Not worried at all (1)** | **b. Not too worried (2)** | **c. Unsure (3)** | **d. More worried (4)** | **e. Very worried (5)** |
| **5. Are you worried that constipation may affect the health of the fetus in the later stages of pregnancy?** | 79(15.3) | 80(15.5) | 127(24.6) | **140(27.1)** | 91(17.6) |
|  | **a. Not important, constipation during pregnancy is difficult to prevent (1)** | **b. Not very important, has little impact on avoiding constipation during pregnancy (2)** | **c. Unsure (3)** | **d. More important, can play a role in avoiding constipation during pregnancy (4)** | **e. Very important, effective in avoiding constipation during pregnancy (5)** |
| **6. Do you think preventive measures (managing your diet and lifestyle) are essential and effective in avoiding constipation in pregnancy?** | 2(0.4) | 7(1.4) | 33(6.4) | **239(46.2)** | 236(45.6) |
|  | **a.Lactulose** | **b.Probiotics** | **c.Laxatives** | **d.Senna leaf** | **e.Only want to improve through diet** |
| **7. For constipation in pregnancy, which treatment or improvement methods would you be willing to consider?** | **312(60.3)** | 373(72.1) | 131(25.3) | 27(5.2) | 304(58.8) |
|  | **a. Strongly disagree (1)** | **b. Disagree (2)** | **c. Unsure (3)** | **d. Agree (4)** | **e. Strongly agree (5)** |
| **8. Do you believe that regulating your mood (avoiding stress and anxiety) can improve constipation symptoms?** | 24(4.6) | 24(4.6) | 162(31.3) | **190(36.8)** | 117(22.6) |

**Supplementary table 3 Distribution of Practice dimension**

|  | **a.Mostly sitting or lying down every day (1)** | **b.Most of the time sitting or lying down (2)** | | | **c.Unsure (3)** | | **d.Occasionally going for a walk (4)** | | | **e.Engaging in regular exercise every day (5)** |
| --- | --- | --- | --- | --- | --- | --- | --- | --- | --- | --- |
| **1. Do you often sit or lie down during pregnancy?** | 46(8.9) | **160(30.9)** | | | 10(1.9) | | 143(27.7) | | | 158(30.6) |
|  | **a.Never stay up late (5)** | **b.Rarely stay up late (4)** | | | **c.Unsure (3)** | | **d.Occasionally stay up late (2)** | | | **e.Frequently stay up late (1)** |
| **2. Do you stay up late during pregnancy?** | 110(21.3) | 100(19.3) | | | 68(13.2) | | **165(31.9)** | | | 74(14.3) |
|  | **a.Never (5)** | **b.Rarely (4)** | | | **c.Unsure (3)** | | **d.Occasionally (2)** | | | **e.Frequently (1)** |
| **3. Do you consume spicy and pungent foods (such as hot pot or spicy hotpot)?** | 88(17.0) | 126(24.4) | | | 51(9.9) | | **201(38.9)** | | | 51(9.9) |
|  | **a.Never (5)** | **b.Rarely (4)** | | | **c.Unsure (3)** | | **d.Occasionally (2)** | | | **e.Frequently (1)** |
| **4. Do you often consume sweet foods (desserts, pastries, etc.)?** | 53(10.3) | 159(30.8) | | | 48(9.3) | | **201(38.9)** | | | 56(10.8) |
|  | **a.Never (1)** | **b.Rarely (2)** | | | **c.Occasionally (3)** | | **d.Frequently (4)** | | | **e.Every meal (5)** |
| **5. Do you consume staple foods (rice, noodles, etc.)?** | 2(0.4) | 32(6.2) | | | 58(11.2) | | 182(35.2) | | | **243(47.0)** |
|  | **a.Never (1)** | **b.Rarely (2)** | | | **c.Unsure (3)** | | **d.Occasionally (4)** | | | **e.Frequently (5)** |
| **6. Do you often eat green vegetables during pregnancy?** | 1(0.2) | 21(4.1) | | | 27(5.2) | | 95(18.4) | | | **373(72.1)** |
|  | **a.Never (5)** | **b.Rarely (4)** | | | **c.Unsure (3)** | | **d.Occasionally (2)** | | | **Frequently (1)** |
| **7. Do you substitute fruits for vegetables in your diet?** | **212(41.0)** | 136(26.3) | | | 51(9.9) | | 87(16.8) | | | 31(6.0) |
|  | **a.Never (5)** | **b.Rarely (4)** | | | **c.Unsure (3)** | | **d.Occasionally (2)** | | | **Frequently (1)** |
| **8. Do you replace plain water with beverages, fruit juices, or milk for drinking?** | **289(55.9)** | 114(22.1) | | | 28(5.4) | | 67(13.0) | | | 19(3.7) |
|  | **a.Never** | **b.Rarely** | | | **c.Unsure** | | **d.Occasionally** | | | **e.Frequently** |
| **9. Do you consume steamed or boiled foods during pregnancy?** | 42(8.1) | 107(20.7) | | | 49(9.5) | | **214(41.4)** | | | 105(20.3) |
|  | **a.No preference** | | | **b.Prefer meat ( meat. poultry. eggs. seafood)** | | | | **c.Prefer vegetarian (leafy greens, legumes, fruits, and root vegetables)** | | |
| **10. Do you have dietary preferences?** | **268(51.8)** | | | 89(17.2) | | | | 160(30.9) | | |
|  | **a.Red meat (pork, beef, lamb, etc.)** | | **b.River/seafood (fish, shrimp, etc.)** | | | **c.Poultry (chicken, duck, goose, etc.)** | | | **d.Eggs and dairy (chicken eggs, dairy products, etc.)** | |
| **10.1 If you prefer meat diet, which type of meat do you often consume? (n=89)** | **67(75.3)** | | 56(62.9) | | | 53(59.6) | | | 64(71.9) | |
|  | **a.Leafy green vegetables (cabbage, oilseed rape, celery, spinach, etc.)** | | **b.Legumes and legume products (soybeans, fava beans, green beans, red beans, etc.)** | | | **c.Melons and fruits (tomatoes, cucumbers, squash, etc.)** | | | **d.Root vegetables (potatoes, sweet potatoes, taro, etc.)** | |
| **10.2 If you prefer a vegetarian diet, which type of vegetarian food do you often consume? (n=160)** | **141(88.1)** | | 69(43.1) | | | 119(74.4) | | | 101(63.1) | |
|  | **a.Three meals at fixed times and in fixed quantities (5)** | **b.Three meals at fixed times but variable quantities (4)** | | | **c.Three meals in fixed quantities but at variable times (3)** | | **d.Frequent extra meals in addition to the three meals (2)** | | | **e.Not fixed times and quantities, eat when you're hungry (1)** |
| **11. Do you have regular eating habits?** | **215(41.6)** | 136(26.3) | | | 43(8.3) | | 66(12.8) | | | 57(11.0) |
|  | **a.Don't pay attention (1)** | **b.Rarely pay attention (2)** | | | **c.Unsure (3)** | | **d.Occasionally pay attention (4)** | | | **Frequently pay attention (5)** |
| **12. Do you pay attention to your bowel movements during pregnancy (volume, frequency, whether the bowel movement is easy to pass, etc.)?** | 6(1.2) | 31(6.0) | | | 35(6.8) | | 150(29.0) | | | **295(57.1)** |
|  | **a.Don't use a phone or read (5)** | **b.Rarely use a phone or read (4)** | | | **c.Unsure (3)** | | **d.Frequently use a phone or read (2)** | | | **e.Almost always use a phone or read (1)** |
| **13. Do you use your phone or read when having a bowel movement?** | 68(13.2) | 62(12.0) | | | 44(8.5) | | **189(36.6)** | | | 154(29.8) |
|  | **a.Don't exceed 10 minutes (5)** | **b.Rarely exceeds 10 minutes (4)** | | | **c.Unsure (3)** | | **d.Frequently exceeds 10 minutes (2)** | | | **Almost always exceeds 10 minutes (1)** |
| **14. Does your time spent on bowel movements exceed 10 minutes?** | 111(21.5) | **143(27.7)** | | | 86(16.6) | | 106(20.5) | | | 71(13.7) |
|  | **a.Never (5)** | **b.Rarely (4)** | | | **c.Unsure (3)** | | **d.Frequently (2)** | | | **e.Almost always (1)** |
| **15. Do you hold or delay having a bowel movement when you feel the urge to have a bowel movement?** | **222(42.9)** | 178(34.4) | | | 64(12.4) | | 38(7.4) | | | 15(2.9) |
|  | **a.Consultation at a hospital** | **b.Consultation at a pharmacy** | | | **c.Online consultation** | | **d.Seek advice from people around you** | | | **Choose dietary self-treatment** |
| **16. If you experience constipation in pregnancy, which of the following ways to seek medical advice or treatment would you choose?** | **318(61.5)** | 41(7.9) | | | 140(27.1) | | 209(40.4) | | | 344(66.5) |
|  | **a.Never (1)** | **b.Rarely (2)** | | | **c.Unsure (3)** | | **d.Usually (4)** | | | **e.Almost always (5)** |
| **17. When experiencing constipation in pregnancy, do you actively regulate your emotions?** | 13(2.5) | 36(7.0) | | | 84(16.2) | | **246(47.6)** | | | 138(26.7) |

**Supplementary table 4 Correlation analysis**

|  | **Knowledge** | **Attitude** | **Practice** |
| --- | --- | --- | --- |
| **Knowledge** | 1.000 | / | / |
| **Attitude** | 0.150(P＜0.001) | 1.000 | / |
| **Practice** | 0.039(P=0.381) | -0.049(P=0.268) | 1.000 |
